# A New Variance-Covariance Matrix for Improving Positioning Accuracy in High-Speed GPS Receivers

**DOI:** 10.3390/s21217324

**Published:** 2021-11-03

**Authors:** Narjes Rahemi, Mohammad Reza Mosavi, Diego Martín

**Affiliations:** 1 ETSI de Telecomunicación, Universidad Politécnica de Madrid, Av. Complutense 30, 28040 Madrid, Spain; rahemi@elec.iust.ac.ir; 2 Department of Electrical Engineering, Iran University of Science and Technology, Tehran 13114-16846, Iran; m_mosavi@iust.ac.ir

**Keywords:** GPS positioning, EKF, UKF, dynamic model, VCM

## Abstract

One of the main challenges in using GPS is reducing the positioning accuracy in high-speed conditions. In this contribution, by considering the effect of spatial correlation between observations in estimating the covariances, we propose a model for determining the variance–covariance matrix (VCM) that improves the positioning accuracy without increasing the computational load. In addition, we compare the performance of the extended Kalman filter (EKF) and unscented Kalman filter (UKF) combined with different dynamic models, along with the proposed VCM in GPS positioning at high speeds. To review and test the methods, we used six motion scenarios with different speeds from medium to high and examined the positioning accuracy of the methods and some of their statistical characteristics. The simulation results demonstrate that the EKF algorithm based on the Gauss–Markov model, along with the proposed VCM (based on the sinusoidal function and considering spatial correlations), performs better and provides at least 30% improvement in the positioning, compared to the other methods.

## 1. Introduction

One way to solve the position equations in GPS receivers is to use the Kalman filter (KF), which is a common estimation algorithm. When the observation or transition model is nonlinear, instead of an ordinary KF, the extended Kalman filter (EKF) or unscented Kalman filter (UKF) should be used. The EKF uses the first-order linearization of the nonlinear model of the system based on the Jacobian matrices to propagate the mean and covariance. This may lead to performance degradation, due to the linearization process and mismodeling of the system. In the UKF, a minimal set of sample points around the mean are selected by a deterministic sampling approach. They are then propagated through the nonlinear functions to obtain a new mean and covariance estimate. It is important to note that in the UKF approach, the rounding errors of numerical calculation may lead to the loss of asymmetry and the non-negativity of the covariance matrix. Thus, the convergence rate of the UKF is slow, and the system may be unstable [1].

The UKF is a more operational method than EKF [2]. On the other hand, the gain matrix of the UKF process cannot be controlled in time according to the filtering effect of system covariance. So, the estimated value of the filter cannot fast-track the status of the system [3]. Therefore, in the situations where the receiver moves at high speed, this method is not able to quickly track the position.

In order to ensure proper operation of the KF in the positioning of an object, it is necessary to give it a suitable dynamic model that precisely describes the movement of the object. The KF is highly dependent on the quality of the motion model to prepare a priori knowledge of system dynamics. In real applications, due to unknown motor behaviors, such as accelerating, turning and maneuvering, the vehicle’s dynamics are usually unknown, making it challenging to create a reasonable dynamic model. Choosing an inappropriate model, especially when the GPS observations are distorted, due to phenomena such as signal interruption, multi-path, etc., can lead to a reduced filter performance or even divergence [4]. So far, different models have been proposed to describe the motion of an object, such as the constant velocity or acceleration models, white noise models, Markov and semi-Markov process models, current statistical model, Autoregressive (AR) process models, and so on [5,6,7]. The multiple model approach has been proposed for target tracking, which is a successful method; but this method leads to a significant computational load, and to use it, its computational requirements must be considered [7,8].

Commonly used methods usually have model biases that make them different from real dynamics. There are two main strategies to counteract these biases. The first strategy assumes that the model biases are subject to random error and therefore, compensates for them in the stochastic model, where the variances of the observations are added to the KF algorithm. Such methods balance the assistance of the observation model and the dynamic model in the navigation solution by reducing inaccurate a priori knowledge. In the other strategy, the motion model bias is added to the state vector to be estimated recursively [4]. This method may lead to an unreliable solution and also a heavy computational load. In this paper, we carry out the first approach.

As mentioned above, to further decrease the positioning error, it is necessary to define the stochastic model precisely [9,10]. The stochastic model represents the statistical characteristics of GPS observables using a variance–covariance matrix, and its misspecification leads to unreliable and non-optimal estimates. The stochastic models for GPS observations can be divided into three general categories: (1) equal-weight models in which the identical variances are selected, (2) elevation-based models in which the weighting of observations is performed, using exponential or trigonometric functions, and (3) SNR-based models in which the weighting of observations depends on the SNR values [11].

The use of diagonal covariance matrices in which the correlations between GPS measurements are ignored leads to unreliable positioning results. Considering the correlations, it causes the off-diagonal elements of the VCM to be non-zero. So, a proper stochastic model for GPS observations should depend on both the satellite elevation angle and the possible correlations of GPS observations [12].

So far, various methods have been proposed to determine the VCM. In these methods, the VCM matrix is either defined as diagonal, which results in less accurate positioning, or is defined as fully populated, which results in an excessive increase in the computational load in each iteration of the positioning algorithm [11,13,14,15].

In this paper, we compare the performance of EKF and UKF methods in the positioning of high-speed GPS receivers and also examine their positioning accuracy when using different dynamic models. Then, given that we are looking for a way to determine the position of a high-speed target, using pseudorange observations and a single-frequency GPS receiver, we propose a VCM according to the above conditions and away from any additional processing load in a way that not only improves positioning accuracy, but also does not impose a high computational cost on the system. The goal of this contribution is to decrease and eliminate the positioning error and improve the reliability and accuracy of the navigation solution in high-speed conditions without significantly increasing the computational complexity. Finally, based on the tests performed on different motion scenarios with different speeds in a wide range from 100 m/s to 7000 m/s, we compare the performance of different positioning methods and examine the effect of the proposed VCM on their accuracy. The simulation results show that the proposed VCM leads to an improvement of at least 30% in the performance of positioning methods.

This paper is organized as follows. Section 2 describes the solution of the navigation equations, using EKF and UKF. Section 3 briefly introduces some dynamic models. In Section 4, the proposed VCM is presented. Section 5 presents the simulation results. Section 6 discusses the results. Finally, in Section 7, the conclusion is given.

## 2. Solving Navigation Equations

### 2.1. Extended Kalman Filter Algorithm

The KF is one of the most important and common estimation algorithms. It was introduced by Rudolf E. Kalman in 1960. The KF is an algorithm that uses a series of observations containing statistical noise and other inaccuracies over time to generate estimates of unknown variables more accurately than those that only are based on a single measurement [16].

The algorithm has two steps. In the first step, the KF generates estimates of current state variables along with their error covariance. When the subsequent measurement is observed (including some errors such as random noise), these estimates are updated using a weighted average, giving more weight to estimates with higher certainty [17]. The algorithm is recursive and does not need to store previous information; so, it is suitable for real-time applications.

The following equations describe a general nonlinear system:

- The state transition model:
  (1) $$x_{k}=f_{k-1}\left(x_{k-1}\right)+w_{k}$$
- The measurement model:
  (2) $$z_{k}=h_{k}\left(x_{k}\right)+v_{k}$$
  where $x_{k}$ is the state vector and $z_{k}$ is the measurement vector at $t_{k}$. $v_{k}$ and $w_{k}$ are the measurement noise vector and the process noise vector at $t_{k}$; both of them are supposed to be zero-mean, white, additive, and independent of each other with covariance $R_{k}$ and $Q_{k}$. In addition, $f_{k-1}$ is the state transition matrix, which is a nonlinear function, and $h_{k}$ is the measurement matrix.

The EKF algorithm for obtaining the optimal estimate of $x_{k}$ is as follows:

- Prediction:
  (3) $${\hat{x}}_{k|k-1}=f_{k-1}\left({\hat{x}}_{k-1|k-1}\right)$$
  (4) $$P_{k|k-1}=F_{k}P_{k-1|k-1}F_{k}^{T}+Q_{k}$$
- Estimation:
  (5) $$K_{k}=P_{k|k-1}H_{k}^{T}{\left[H_{k}P_{k|k-1}H_{k}^{T}+R_{k}\right]}^{-1}$$
  (6) $$P_{k|k}=\left(I-K_{k}H_{k}\right)P_{k|k-1}$$
  (7) $${\hat{x}}_{k|k}={\hat{x}}_{k|k-1}+K_{k}\left[z_{k}-h_{k}\left({\hat{x}}_{k|k-1}\right)\right]$$
  where $F_{k}$ and $H_{k}$ are the Jacobian matrices of $f_{k}\left(.\right)$ and $h_{k}\left(.\right)$, $P_{k|k}$ is the error covariance at $t_{k}$, and $K_{k}$ is the near-optimal Kalman gain.

### 2.2. Unscented Kalman Filter Algorithm

The UKF is an extension of unscented transform (UT) to the KF. The UT is an efficient sigma point sampling technique that picks a minimal set of sample points, which is called sigma points, around the mean and then propagates them through nonlinear functions. The result is a new mean and covariance estimate. In this method, which set of sigma points are selected and how the converted statistics of the UT are calculated are vital.

When the state transition and observation functions are highly nonlinear, the EKF can provide poor performance because the covariance is propagated through the linearization of the nonlinear model [18]. In terms of computational complexity, UKF is the same as EKF [2].

There are three types of sampling strategies for UT: (1) simplex, (2) symmetric, and (3) scaled. The scaled type allows some weighting adjustments to improve robustness against higher-order non-linearities beyond second order. It uses three adjustable scaling parameters $\left(\alpha ,\beta ,\lambda \right)$ to allow for some tuning required by the specific applications [2]. The UKF algorithm based on the scaled strategy is as follows:

**A.**  **Prediction:**
  Consider a random variable *x*:
  (1) Compute the set of sigma points in state space:
    (8) $$\left\{\begin{array}{c} \chi_{i}=\mu_{x}+\sqrt{\left(n+\lambda \right)c_{i}}\\ \chi_{n+i}=\mu_{x}-\sqrt{\left(n+\lambda \right)c_{i}} \end{array}\right.,\;1\leq i\leq n$$
    (9) $$\chi_{0}=\mu_{x}$$
    (10) $$\lambda =\alpha^{2}\kappa +\left(1-\alpha^{2}\right)n$$
    where χ is the sigma points matrix, $\mu_{x}$ is the mean in state space, c is the covariance matrix, *n* is the dimension of the system, λ is the scaling factor, κ is the second scaling factor, and α determines the spread of the sigma points around the mean.
  (2) Calculate the weight of each sigma point:
    (11) $$W_{i}=\frac{1}{2n+2\lambda },\;1\leq i\leq 2n$$
    (12) $$W_{0}=\frac{\lambda }{n+\lambda }$$
    where W is the weight matrix.
  (3) Transform the sigma points through the nonlinear function:
    (13) $$\xi_{i}=f\left(\chi_{i}\right),\;0\leq i\leq 2n$$
    where f is the nonlinear state transition function and ξ is the transformed sigma points matrix.
  (4) The approximate new mean and covariance are as follows:
    (14) $$\mu_{x}^{\prime }=W_{0}\xi_{0}+\overset{2n}{\underset{i=1}{\sum }}W_{i}\xi_{i}$$
    (15) $$P_{xx}^{\prime }=W_{0}^{\left[P_{xx}^{\prime }\right]}\left(\xi_{0}-\mu_{x}^{\prime }\right){\left(\xi_{0}-\mu_{x}^{\prime }\right)}^{T}+\overset{2n}{\underset{i=1}{\sum }}W_{i}(\xi_{i}-\mu_{x}^{\prime }){\left(\xi_{i}-\mu_{x}^{\prime }\right)}^{T}+Q$$
    (16) $$W_{0}^{\left[P_{xx}^{\prime }\right]}=\frac{\lambda }{n+\lambda }+1-\alpha^{2}+\beta$$
    where $\mu_{x}^{\prime }$ is the predicted mean, $P_{xx}^{\prime }$ is the predicted covariance, Q is the process noise covariance, and β is used to incorporate prior knowledge of the distribution of *x* [19].
**B.**  **Estimation:**
  (5) The update step considering measurements is as follows:
    (17) $${Ζ}_{i}=h\left(\chi_{i}\right),\;0\leq i\leq 2n$$
    (18) $$\mu_{z}^{\prime }=\overset{2n}{\underset{i=0}{\sum }}W_{i}{Ζ}_{i}$$
    (19) $$P_{zz}=\overset{2n}{\underset{i=0}{\sum }}W_{i}\left({Ζ}_{i}-\mu_{z}^{\prime }\right){\left({Ζ}_{i}-\mu_{z}^{\prime }\right)}^{T}+R$$
    where h is the nonlinear measurement function, Ζ is the transformed sigma points in measurement space, $\mu_{z}^{\prime }$ is the predicted mean in measurement space, $P_{zz}$ is the predicted covariance in measurement space, and R is the measurement noise covariance.
  (6) Calculate the cross-covariance and Kalman gain as follows:
    (20) $$P_{xz}=\overset{2n}{\underset{i=0}{\sum }}W_{i}\left({Ζ}_{i}-\mu_{z}^{\prime }\right){\left(\xi_{0}-\mu_{x}^{\prime }\right)}^{T}$$
    (21) $$K=P_{xz}P_{zz}{}^{-1}$$
  (7) Perform the update as follows:
    (22) $$\mu_{x}=\mu_{x}^{\prime }+K\left(z-\mu_{z}^{\prime }\right)$$
    (23) $$c=P_{xx}^{\prime }-KP_{zz}K^{T}$$
    where z is the observation matrix.

## 3. The Dynamic Model

Dynamic models describe the state trajectory of a target. The target, which can be different types of vehicles, such as cars, aircraft, ships, etc., performs various kinds of maneuvers. As a result, a simple model may not represent all types of maneuvers precisely, and particular models are needed to describe and predict the trajectory according to each application. These motion models are used in the state transition matrix of filtering methods, such as the KF. With the aim of motion models, the KF can predict the next value in target tracking. The better the models are, the better the performance of the filter. So, it is essential to study and select an appropriate model [20].

The movement of objects is classified into two types: non-maneuvers and maneuvers. The non-maneuvering motion is moving on a straight line at a constant speed. Other types of movements fall into the category of maneuver. In this paper, by selecting three models from the maneuver category, i.e., white noise models, Markov process models, and AR process models, and applying them to the EKF and UKF algorithms, we examine their performance in positioning the objects at high speeds.

### 3.1. White Noise Models

**Figure 1:**
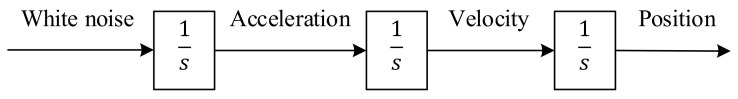
White noise model [21].

In these models, the control input of the system is considered white noise. Here, we have focused on the Wiener process acceleration model. It assumes that the acceleration is a Wiener process or a process with independent increments, which is not necessarily a Wiener process. In other words, this model supposes that jerk is an independent process (white noise). It is also known as the nearly-constant-acceleration model [6]. The block diagram of the Wiener process acceleration model is presented in Figure 1. In this model, the transition matrix (F) and the process noise covariance matrix (Q), which is introduced in Section 2.1, for $x={\left[position,velocity,acceleration\right]}^{\prime }$, are as follows:

(24) $$F=\left[\begin{array}{ccc} 1 & T & T^{2}/2\\ 0 & 1 & T\\ 0 & 0 & 1 \end{array}\right]$$

(25) $$Q=\left[\begin{array}{ccc} T^{5}/20 & T^{4}/8 & T^{3}/6\\ T^{4}/8 & T^{3}/3 & T^{2}/2\\ T^{3}/6 & T^{2}/2 & T \end{array}\right]$$

where *T* is the time interval between samples.

### 3.2. Markov Process Models

**Figure 2:**
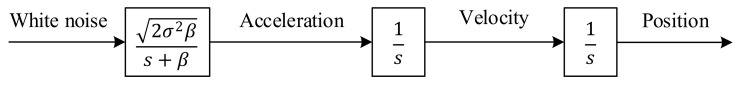
Gauss–Markov model [21].

These models assume the acceleration of the target as a Markov process. Among these models, we focus on the Singer model, in which the acceleration is a zero-mean first-order Gauss–Markov process with variance $\sigma^{2}$ and time constant 1β. The block diagram of the Gauss–Markov model is shown in Figure 2. The *F* and *Q* matrices in this model are as follows [21]:

(26) $$F=\left[\begin{array}{ccc} 1 & T & \left(\beta T+e^{-\beta T}-1\right)/\beta^{2}\\ 0 & 1 & \left(1-e^{-\beta T}\right)/\beta \\ 0 & 0 & e^{-\beta T} \end{array}\right]$$

(27) $$Q=\left[\begin{array}{cccc} Q_{p} & 0 & 0 & 0\\ 0 & Q_{p} & 0 & 0\\ 0 & 0 & Q_{p} & 0\\ 0 & 0 & 0 & Q_{c} \end{array}\right]$$

(28) $$Q_{p}=\frac{\sigma^{2}}{\beta^{2}}\left[\begin{array}{ccc} \frac{2T-4T^{2}e^{-\beta T}}{\beta }-\frac{e^{-2\beta T}-1}{\beta^{2}}-2T^{2}+\frac{2\beta T^{3}}{3} & \frac{1}{\beta }{\left(\beta T+e^{-\beta T}-1\right)}^{2} & 1-e^{-2\beta T}-2\beta Te^{-\beta T}\\ \frac{1}{\beta }{\left(\beta T+e^{-\beta T}-1\right)}^{2} & 2\beta T+1-{\left(e^{-\beta T}-2\right)}^{2} & \beta {\left(e^{-\beta T}-1\right)}^{2}\\ 1-e^{-2\beta T}-2\beta Te^{-\beta T} & \beta {\left(e^{-\beta T}-1\right)}^{2} & \beta^{2}\left(e^{-2\beta T}-1\right) \end{array}\right]$$

(29) $$Q_{c}=\left[\begin{array}{cc} S_{f}T+S_{g}T^{3}/2 & S_{g}T^{2}/2\\ S_{g}T^{2}/2 & S_{g}T \end{array}\right]$$

where $S_{f}$ and $S_{g}$ are the spectral amplitudes for the clock model [21].

### 3.3. Autoregressive Process Models

An AR process is a time series generated by a linear combination of past values. It can be described by a linear equation as follows:

(30) $$x\left(n\right)=-\overset{p}{\underset{k=1}{\sum }}\alpha_{k}x\left(n-k\right)+w\left(n\right)$$

where $x\left(n\right)$ is the process output which is a combination of past values plus white noise, $\alpha_{k}$ are the model parameters, and *p* is the order of the AR process [22]. Considering the third-order AR process, we can write the following:

(31) $$\dot{a}\left(t\right)=-\alpha_{3}p\left(t\right)-\alpha_{2}v\left(t\right)-\alpha_{1}a\left(t\right)+w\left(t_{k}\right)$$

where $a\left(t\right)$ is the acceleration, $v\left(t\right)$ is the velocity, and $p\left(t\right)$ is the position. Therefore, the state-space representation is as follows:

(32) $$\dot{x}\left(t\right)=\left[\begin{array}{ccc} 0 & 1 & 0\\ 0 & 0 & 1\\ -\alpha_{3} & -\alpha_{2} & -\alpha_{1} \end{array}\right]x\left(t\right)+\left[\begin{array}{c} 0\\ 0\\ 1 \end{array}\right]w\left(t\right)$$

**Figure 3:**
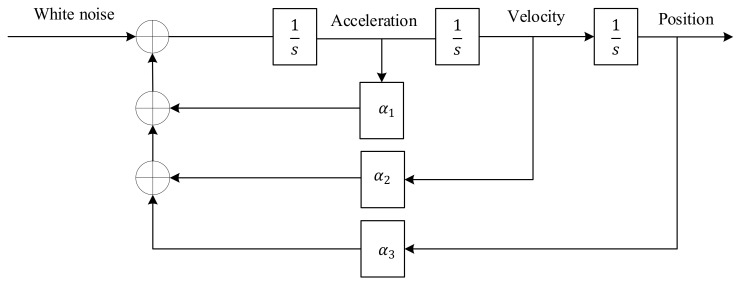
Third-order AR process model.

The block diagram of the third-order AR process model is shown in Figure 3. It is important to note that each increase in the order of the AR model leads to an increase in the variables of the KF by three because the model is applied to each axis. In this paper, we focus on the second-order AR model due to the fact that a higher-order would increase the computational complexity and might lead to unstable solutions. The transition matrix can be obtained from solving the dynamic system’s vectorial differential equation [23].

## 4. Proposed VCM

To create an optimal solution, the KF algorithms combine the measurement and dynamic models. For this purpose, the weights of both models must be adjusted properly. These weights are expressed by stochastic models or, in other words, by their covariance matrices. In the previous sections, the functional models were discussed. In this section, the stochastic model of observations is presented.

Proper weighting of observations is necessary because the GPS measurements at different epochs or from different satellites do not have the same precision. In other words, the quality of observations is different from each other. This is mainly due to factors such as random noise, correlations between measurements, tracking loop characteristics, receiver dynamics, signal strength, multipath and atmospheric effects, and so on. Therefore, by giving a higher weight (or lower variance) to precise observations and contributing them to estimating the parameters more than imprecise ones, while using all observations in positioning, we can reduce the impact of lower quality observations on positioning and consequently achieve higher accuracy.

The quality of observations can be measured, using two signal parameters: (1) signal-to-noise ratio (C/N0) and (2) elevation angle [24]. In [15], the accuracy of positioning using the KF algorithm along with different variance estimation methods by means of these two parameters is investigated. Additionally, by ignoring the correlation between observations, the covariance matrix is defined as diagonal. The physical correlations are not usually considered in the stochastic model of GPS observations. In this paper, we use the commonly used sinusoidal model for variance estimation and consider the correlations between observations; based on it, the non-diagonal covariance matrix is determined.

The physical correlations include three types of spatial, temporal, and cross correlations that describe the observational dependencies in space, over time, and between frequencies, respectively. The temporal correlation, that is, the correlation between observations at different epochs, is generally significant when GPS double difference observations in relative positioning is used. In the case of precise point positioning, less correlation is expected since the double differencing procedure may increase the correlation time of the GPS phase observations [14]. The spatial correlation between observations of different satellites within one epoch in one site is due to the similar observational conditions for those measurements. The cross correlations between the L1 and L2 carriers are considerable, while the correlation between the phase and code observations is negligible [14].

Based on the above explanations and due to the fact that in this work, the non-differential pseudo-range observations are used for positioning, the spatial correlation is considered, and the other correlations are ignored.

Generally, the stochastic model is expressed by the VCM, which determines the accuracy of the precision of the observations by the main elements (variances) and the correlations between observations by the off-diagonal elements (covariances).

As mentioned earlier, in [15], we examined different variance estimation methods and compared the positioning results using various models. Based on these results, the sinusoidal model leads to the best positioning accuracy and the lowest error. Therefore, we use the sine function in the proposed VCM. In this way, the proposed VCM for n satellites at epoch $t_{k}$ is constructed as follows:

(33) $$R_{k}=\left[\begin{array}{cccc} \sigma_{1,1}^{2} & \sigma_{1,2} & \cdots & \sigma_{1,n}\\ \sigma_{2,1} & \sigma_{2,2}^{2} & \; & \vdots \\ \vdots & \; & \ddots & \;\\ \sigma_{n,1} & \cdots & \; & \sigma_{n,n}^{2} \end{array}\right]$$

(34) σi,i2=1sin2ELitk

where $\sigma_{i,i}^{2}$ is the variance of observations from satellite *i*. The spatial correlation coefficient, which shows the observational dependencies in space, is expressed as follows:

(35) $$\rho_{i,j}=\frac{\sigma_{i,j}}{\sqrt{\sigma_{i,i}^{2}\;\sigma_{j,j}^{2}}}$$

where $\sigma_{i,j}$ is the covariance between observations from satellite *i* and satellite *j*. Therefore, the covariance of the observations can be calculated as follows:

(36) σi,j=δsinELitksinELjtk,    i,j=1,…,n

where $EL_{i}\left(t_{k}\right)$ represents the elevation angle of satellite *i* at epoch $t_{k}$ and $\delta \epsilon \left[0,1\right]$ is a constant factor.

## 5. Simulation Results

Raw GPS data, such as pseudo-range, carrier phase, satellite ephemeris, Doppler shift, etc., are generated using Rohde & Schwarz GNSS simulator. All GNSS signals can be generated in real time using this simulator, taking into account the signal propagation, user environment, and the system characteristics, such as (1) orbit errors, clock errors, atmospheric effects, (2) signal obscuration and multipath, (3) vehicle motion and vehicle attitude, (4) antenna characteristics and so on.

**Figure 4:**
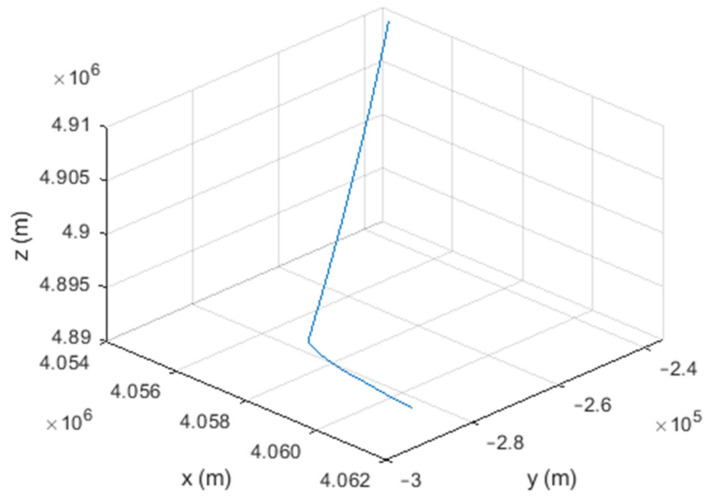
The trajectory of the first scenario: moving at a speed of 90 m/s.

**Figure 5:**
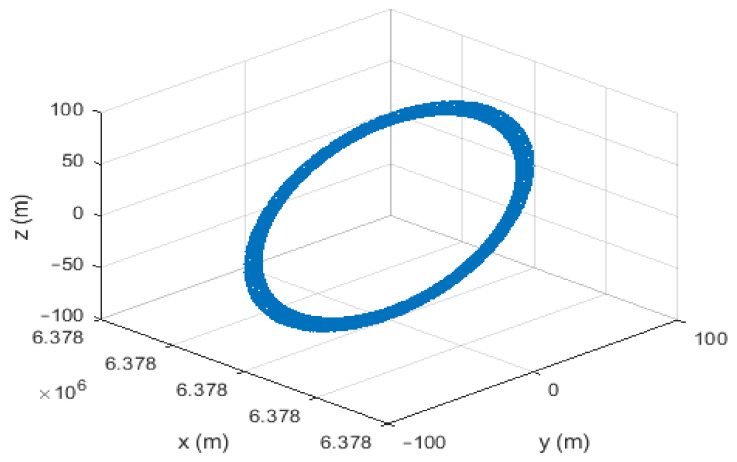
The second scenario: moving in a circular path at a speed of 100 m/s.

**Figure 6:**
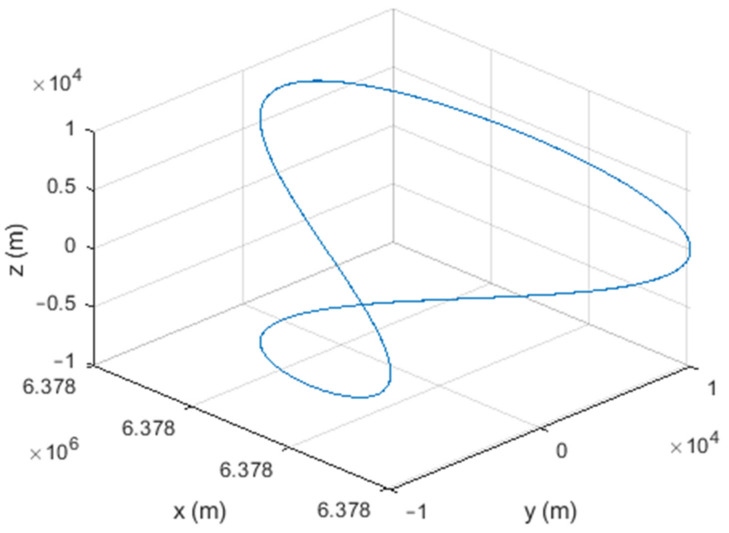
The third scenario: moving in a circular path at a speed of 500 m/s.

**Figure 7:**
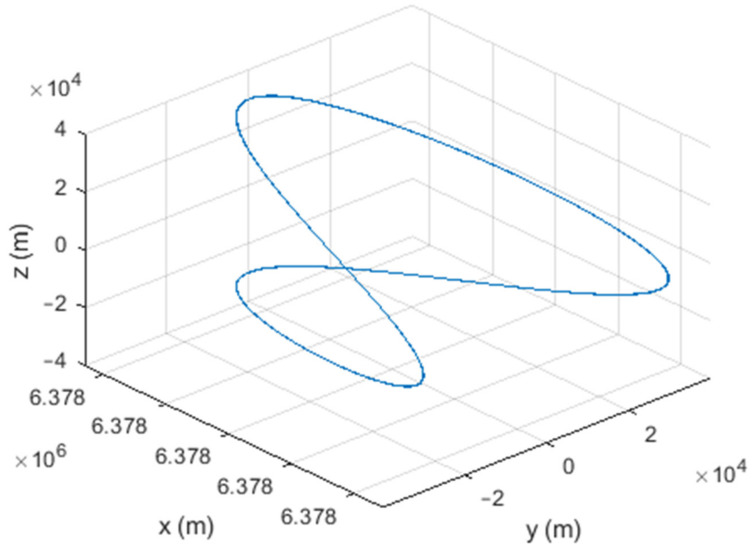
The fourth scenario: moving in a circular path at a speed of 3500 m/s.

**Figure 8:**
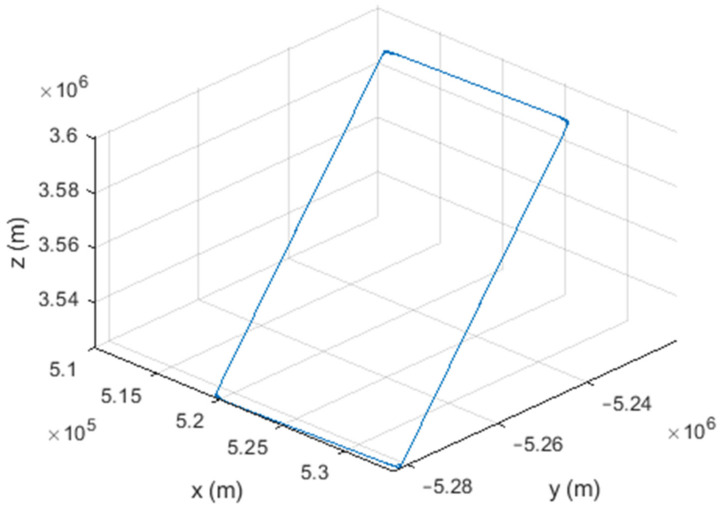
The fifth scenario: moving in a rectangular path at a speed of about 3200 m/s.

**Figure 9:**
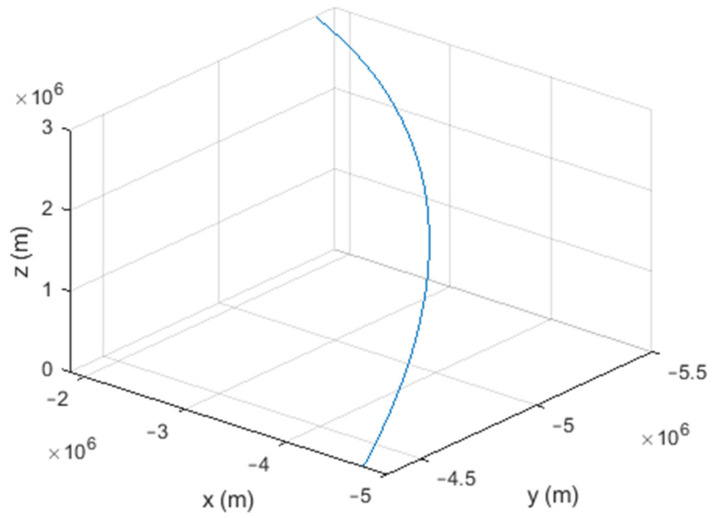
The sixth scenario: moving at a speed of about 7200 m/s in space.

**Figure 10:**
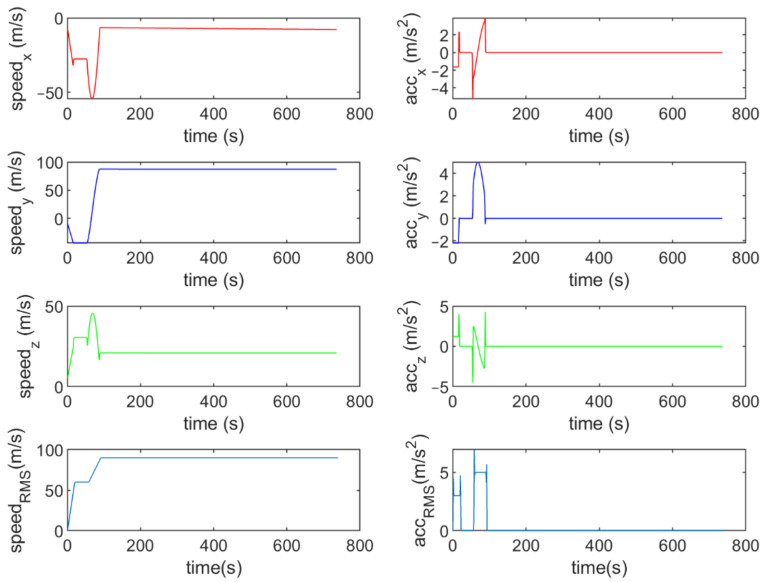
The speed and acceleration components in the first scenario.

**Figure 11:**
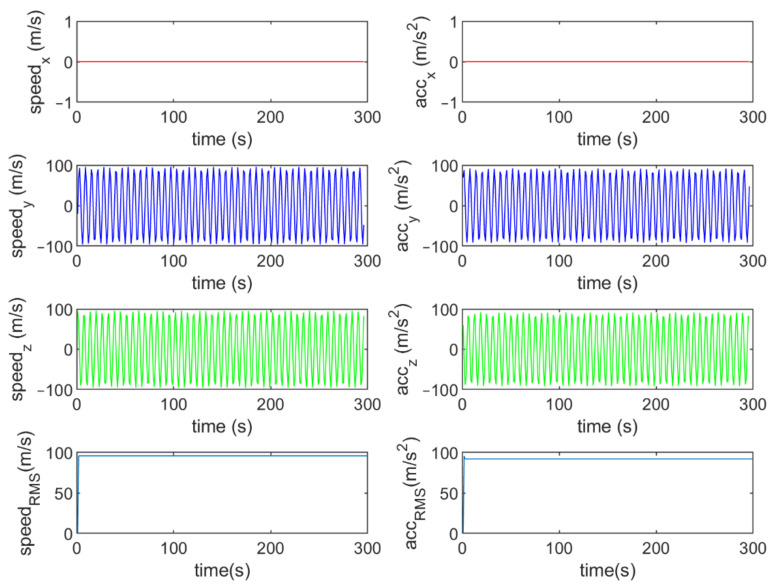
The speed and acceleration components in the second scenario.

**Figure 12:**
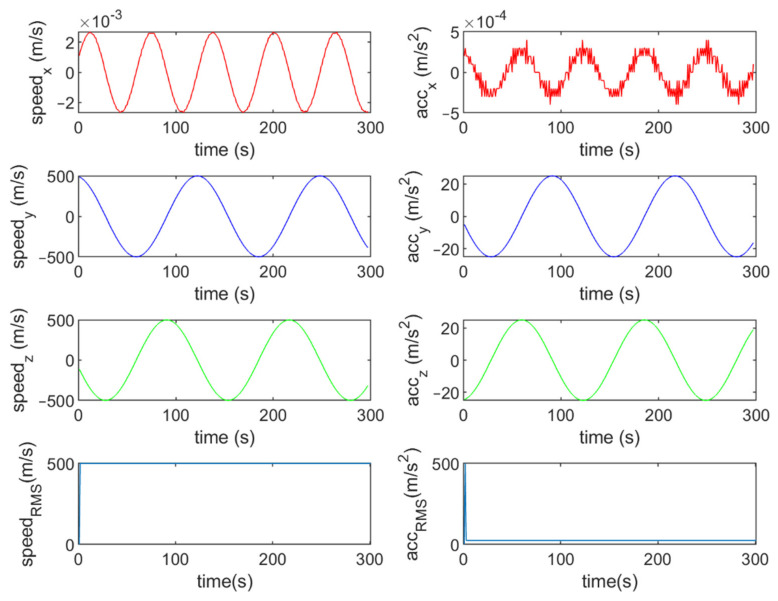
The speed and acceleration components in the third scenario.

**Figure 13:**
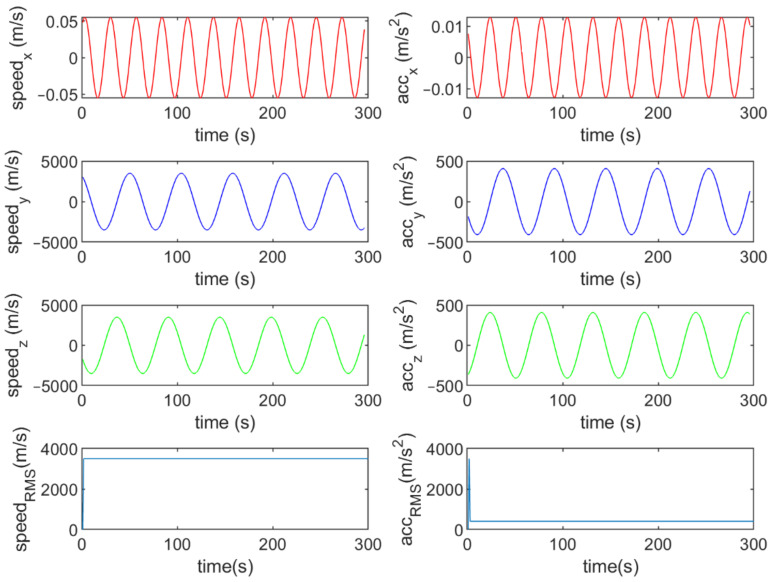
The speed and acceleration components in the fourth scenario.

**Figure 14:**
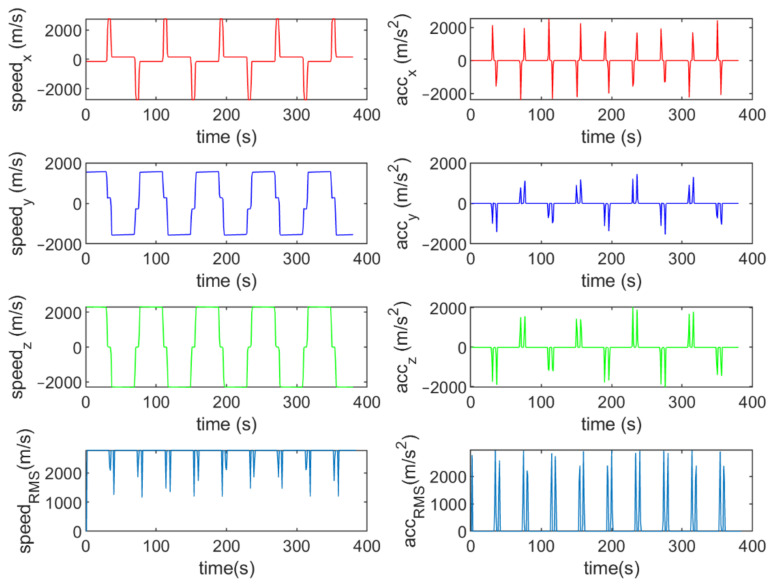
The speed and acceleration components in the fifth scenario.

**Figure 15:**
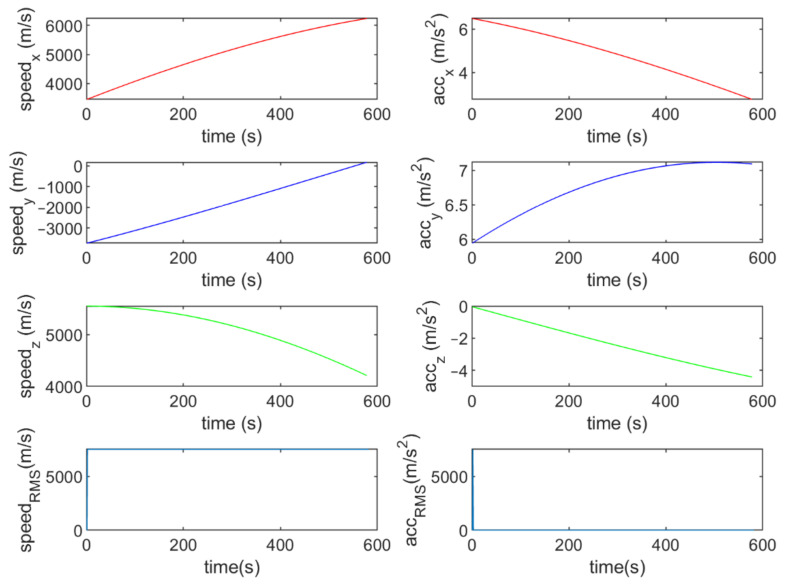
The speed and acceleration components in the sixth scenario.

Six different movement scenarios are used to evaluate the performance of the methods. Test scenarios involve the movement of an airplane with a GNSS receiver inside. The first scenario concerns the movement of an aircraft at a speed of 90 m/s. The second to fourth scenarios are related to moving in a circular path at 100 m/s, 500 m/s, and 3500 m/s. The fifth scenario is moving with a speed of 3200 m/s in a rectangular path. The last scenario is moving at a speed of about 7200 m/s in space. The trajectories of the scenarios and their speeds and accelerations are shown in Figure 4, Figure 5, Figure 6, Figure 7, Figure 8, Figure 9, Figure 10, Figure 11, Figure 12, Figure 13, Figure 14 and Figure 15.

**Figure 16:**
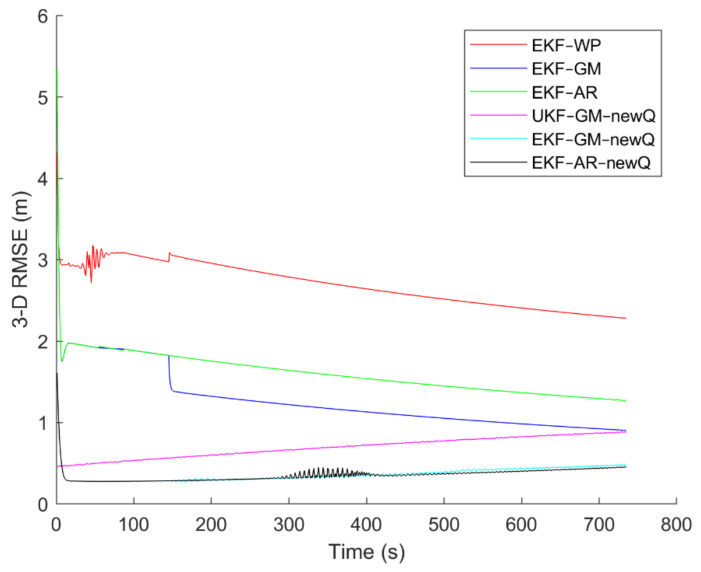
The positioning error of different methods in the first scenario.

**Figure 17:**
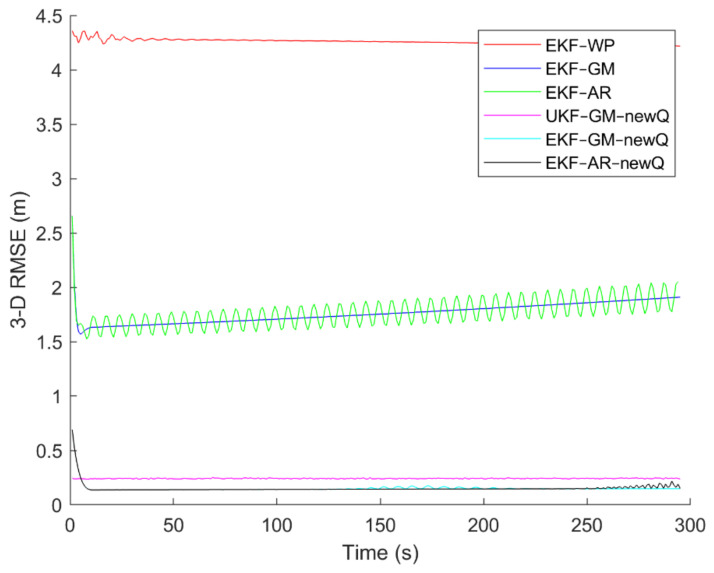
The positioning error of different methods in the second scenario.

**Figure 18:**
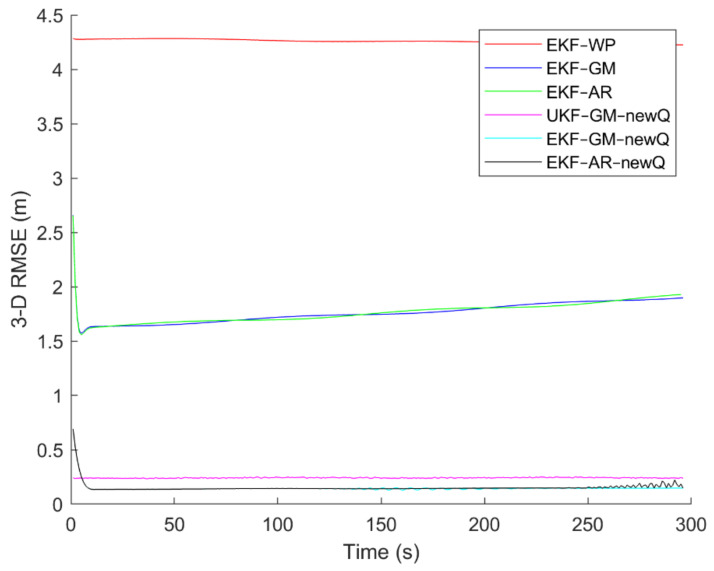
The positioning error of different methods in the third scenario.

**Figure 19:**
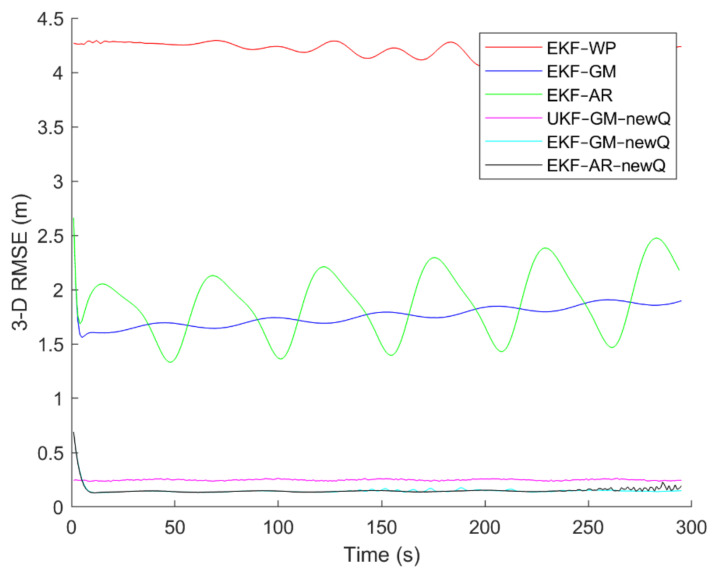
The positioning error of different methods in the fourth scenario.

**Figure 20:**
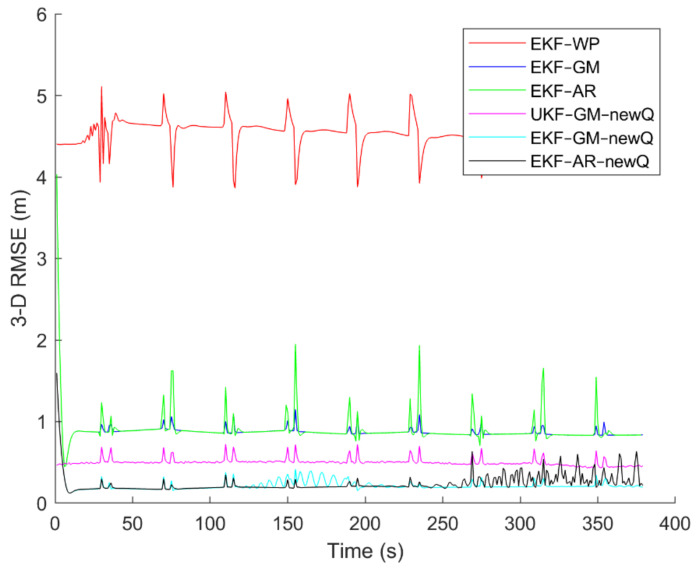
The positioning error of different methods in the fifth scenario.

**Figure 21:**
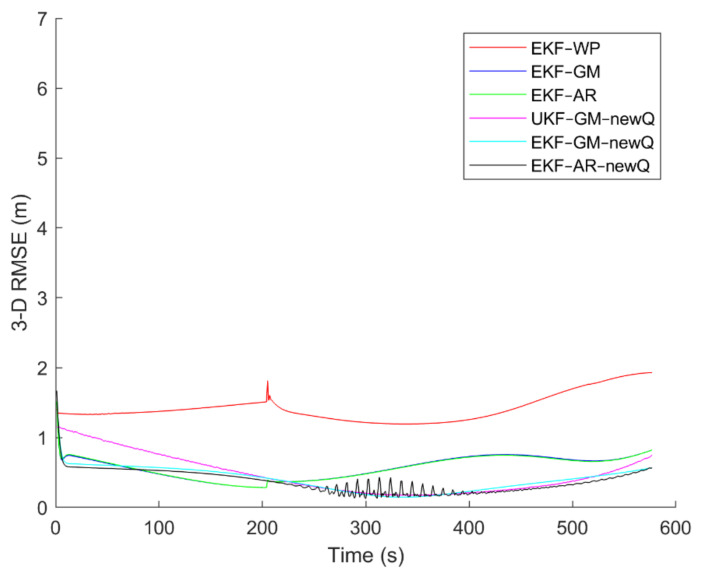
The positioning error of different methods in the sixth scenario.

To evaluate the performance of the methods, positioning was performed, using the EKF and UKF algorithms along with the Wiener process (WP), Gauss–Markov (GM), and AR models, and with diagonal VCM (diagonal Q) and the proposed VCM (new Q). The positioning results are shown in Figure 16, Figure 17, Figure 18, Figure 19, Figure 20 and Figure 21. In addition, the 3D root mean square errors (RMSE) of the positioning methods are presented in Table 1. The parameters used in the AR model are $\alpha_{1}=0.1,\;\alpha_{2}=0.2$, and $\alpha_{3}=0$. Based on these values of parameters, the transition matrix is as shown in Equation (37). We also selected α=0.02, β=2, and λ=0 for the scaled UKF algorithm.

(37) $$F=\left[\begin{array}{ccc} 1 & e^{-0.1T}\left(8/3\sin\left(0.3T\right)-2\cos\left(0.3T\right)+2\right) & 10e^{-0.1T}\left(1-1/3\sin\left(0.3T\right)-\cos\left(0.3T\right)\right)\\ 0 & e^{-0.1T}\left(1/3\sin\left(0.3T\right)+\cos\left(0.3T\right)\right) & 10/3e^{-0.1T}\sin\left(0.3T\right)\\ 0 & -1/3e^{-0.1T}\sin\left(0.3T\right) & e^{-0.1T}\left(\cos\left(0.3T\right)-1/3\sin\left(0.3T\right)\right) \end{array}\right]$$

Table 2 shows the percentage of improvement in the positioning accuracy of the EKF algorithm using the proposed VCM in each scenario. Additionally, Table 3 presents the CPU time of the EKF algorithm to determine the position of a point with and without the proposed VCM in milliseconds.

**Figure 22:**
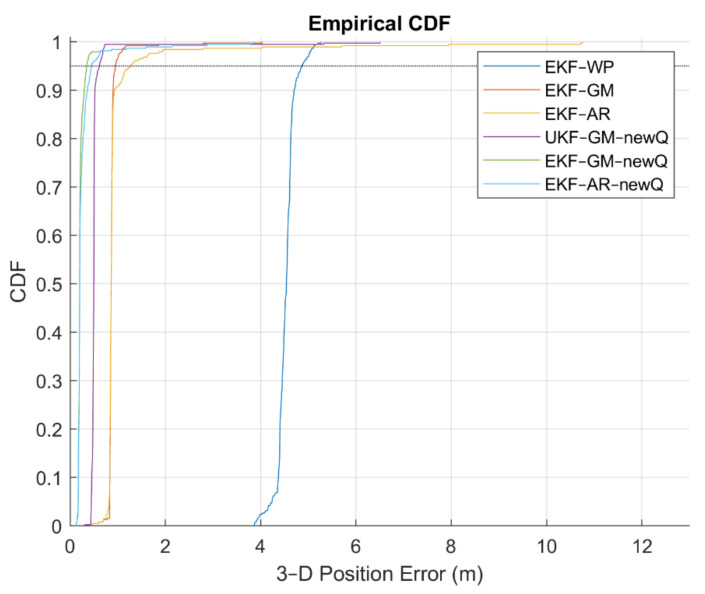
The empirical CDF of the fifth scenario.

**Figure 23:**
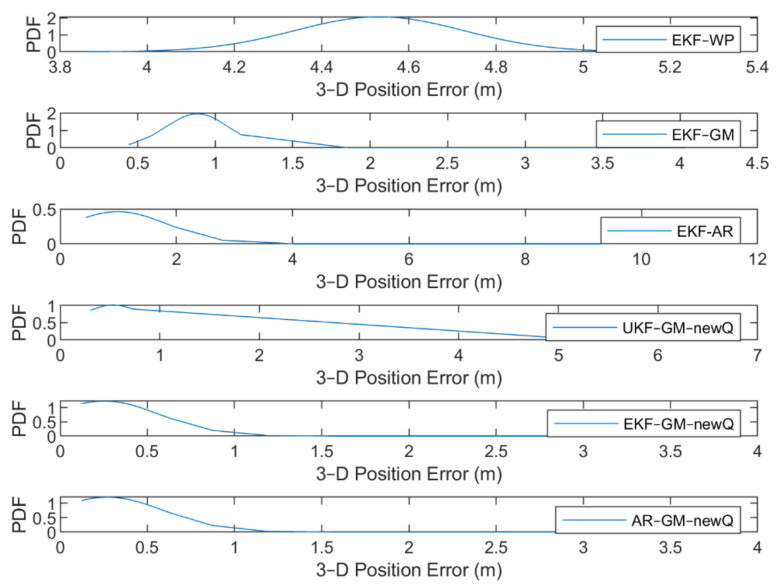
The PDF of the fifth scenario.

To further compare the performance of different methods, the cumulative density functions (CDF) and probability density functions (PDF) of positioning error for the fifth scenario (“Rectangular” data) are given in Figure 22 and Figure 23. Additionally, some statistical characteristics of the positioning results with different methods related to the fifth scenario are presented in Table 4.

## 6. Discussion

It can be seen from Figure 16, Figure 17, Figure 18, Figure 19, Figure 20 and Figure 21 that the error curve of the EKF algorithm with the Gauss–Markov or second-order AR model along with the proposed VCM is always lower than the error curve of other methods. In addition, the number and size of jumps in the error curve of these methods are less than others. Especially in such scenarios as rectangular motion (fifth scenario) in which there are sudden jumps in acceleration (and thus in speed and position), these methods perform better and have a smoother error curve than other methods. There are fewer fluctuations in the error curve of these methods and the probability of divergence is less.

On the other hand, it can be seen that in scenarios in which the error accumulates over time and the error curve is ascending (such as the second to fourth scenarios), the methods that use the proposed VCM do not have incremental error. Therefore, based on the figures, it can be obtained that the EKF algorithm with GM or second-order AR model along with the proposed VCM offers better performance in terms of stability, smoothness and accuracy than other methods.

In terms of positioning accuracy, we can say that the AR model performs slightly better than the GM model; on the other hand, the output is more likely to fluctuate than the GM model because the AR model is completely dependent on the parameters and the slightest mistake in selecting the parameters will lead to output instability and may even lead to system divergence.

According to Table 1, it can be seen that the accuracy of the GM model is better than the Wiener process and the second-order AR models. Additionally, it is observed that the EKF achieves better accuracy than the UKF, and this confirms that the UKF cannot fast-track the system status at high-speed conditions. It is also observed that the proposed VCM in all cases has improved the positioning accuracy of the methods.

Explaining the reason for the differences in the results of scenarios, we can say the following: In the first scenario, the number of observed satellites is 6, while this number for the second to fourth scenarios is 10 and in the last scenario is 8. Therefore, the accuracy of positioning in this scenario, despite being slower, is less than the next four scenarios. In the sixth scenario, in addition to the fact that the number of observed satellites is 8, the acceleration and speed of movement are very high and are very different from the previous scenarios; as a result, the largest amount of positioning error is related to this scenario.

From Table 2, it is observed that the proposed VCM in the last scenario, which has the highest acceleration and speed, results in a 30% improvement and in other scenarios, it makes at least 70% improvements. In addition, from Table 3, it can be seen that the GM and AR models are not different in terms of CPU time. In addition, using the proposed VCM does not increase the CPU time.

Figure 22 shows that the positioning error at 95% confidence level in the EKF algorithm is better than the UKF algorithm. Using the proposed VCM, this value is better than the diagonal VCM. Finally, the best case is the EKF algorithm with the GM or AR model along with the proposed VCM.

Figure 23 shows that the maximum PDF in the EKF algorithm with GM and AR models occurred at a position error of 25 cm and the errors start from a value of about 12 cm. In the EKF-WP, EKF-GM, EKF-AR and UKF-GM new Q methods, the error values start from 3.8 m, 44 cm, 44 cm and 30 cm, and the maximum PDF occur in the values of 4.5 m, 88 cm, 1 m and 49 cm. The PDF curve of the EKF-GM and EKF-AR methods along with the proposed VCM is more concentrated than other methods. The errors of these methods are in the range of 3.8 m, while this value for other methods is about 1.5, 4, 11 and 6.5 m, respectively. Based on the above description, we find that EKF-GM along with the proposed VCM performs better than other methods with less position error and higher error concentration.

In Table 4, we see that in terms of mean error, RMS error and error (95%), this method works much better than other methods, and only in terms of standard deviation, EKF-GM with the diagonal Q matrix performs slightly better than EKF-GM with the proposed VCM.

## 7. Conclusions

Efforts to improve the performance of GPS receivers over time have continued steadily since its launch. One of the blocks whose performance has a direct impact on the positioning accuracy of the receiver is the navigation section. Errors in the system, such as noise of received data, correlations between observations, systematic errors, etc., affect the efficiency of this block and reduce the positioning accuracy. These errors increase as the receiver speeds up. In this paper, by taking into account the effect of spatial correlation between observations in estimating the variance, an idea for determining VCM is given that improves the positioning accuracy. Additionally, the EKF and UKF algorithms along with the Wiener process, Gauss–Markov, and second-order AR models are used to solve the position equations, and the performance of these methods in high-speed motions are compared to each other. Six different high-speed motion scenarios are used for testing. The experimental results show that, firstly, the EKF algorithm is more accurate than the UKF algorithm at high speeds. Secondly, the use of the proposed VCM instead of diagonal VCM improves performance (of both algorithms) in terms of accuracy, stability, and smoothness, and reduces positioning error by at least 30% in the worst case. Additionally, the results show that the second-order AR and GM models are more accurate than the WP model. The second-order AR model is slightly better than the GM model in terms of accuracy, but on the other hand, it is very dependent on the model parameters, and the slightest mistake in determining the parameters can lead to fluctuations in the output. Finally, the results of the simulations with different motion scenarios with a vast range of speeds from 90 m/s to 7000 m/s show that the EKF algorithm with the Gauss–Markov model and using the proposed VCM has the best performance and the least positioning error in a wide range of moving objects. The features of this method include the stability of the error curve, fewer fluctuations in the output, no increase in error over time and less probability of divergence than other methods.

## Figures and Tables

**Table 1:** The positioning error (RMSE) caused by different methods in [m].

Method		EKF + WP	EKF + GM	EKF + AR	UKF + GM	EKF + GM	EKF + AR
	Q	Diagonal Q	Diagonal Q	Diagonal Q	Proposed Q	Proposed Q	Proposed Q
Data							
First scenario		2.69	1.28	1.59	0.71	0.36	0.35
Second scenario		4.25	1.77	1.78	0.24	0.14	0.14
Third scenario		4.25	1.77	1.77	0.24	0.14	0.14
Fourth scenario		4.19	1.77	1.90	0.25	0.14	0.14
Fifth scenario		4.54	0.87	0.90	0.50	0.21	0.24
Sixth scenario		1.43	0.58	0.58	0.51	0.41	0.38

**Table 2:** The percentage of improvement in the positioning accuracy of the EKF algorithm using proposed VCM.

Data	Percentage of Improvement in EKF + GM Algorithm	Percentage of Improvement in EKF + AR algorithm
First scenario	71	80
Second scenario	92	92
Third scenario	92	92
Fourth scenario	92	93
Fifth scenario	75	73
Sixth scenario	29	34

**Table 3:** The CPU time of EKF algorithm to determine the position of a point with and without the proposed VCM [ms].

Method		EKF + GM	EKF + AR	EKF + GM	EKF + AR
	Q	Diagonal	Diagonal	Proposed	Proposed
Data					
First scenario		0.08	0.09	0.09	0.09
Second scenario		0.13	0.13	0.15	0.14
Third scenario		0.13	0.12	0.12	0.12
Fourth scenario		0.13	0.12	0.13	0.13
Fifth scenario		0.11	0.11	0.12	0.12
Sixth scenario		0.09	0.09	0.11	0.10

**Table 4:** Statistical characteristics of different methods in the fifth scenario in [m].

Method		EKF + WP	EKF + GM	EKF + AR	UKF + GM	EKF + GM	EKF + AR
	Q	Diagonal Q	Diagonal Q	Diagonal Q	Proposed Q	Proposed Q	Proposed Q
Indicator							
RMSE		4.54	0.87	0.90	0.50	0.21	0.24
Mean value of error		4.54	0.88	0.86	0.52	0.25	0.27
Standard deviation of error		0.24	0.20	0.90	0.39	0.32	0.32
Error (95%)		4.86	0.95	1.26	0.62	0.35	0.45

## Data Availability

Not applicable.
